# Synthetic 4D-CT of the thorax for treatment plan adaptation on MR-guided radiotherapy systems

**DOI:** 10.1088/1361-6560/ab0dbb

**Published:** 2019-05-23

**Authors:** Joshua N Freedman, Hannah E Bainbridge, Simeon Nill, David J Collins, Marc Kachelrieß, Martin O Leach, Fiona McDonald, Uwe Oelfke, Andreas Wetscherek

**Affiliations:** 1 Joint Department of Physics, The Institute of Cancer Research and The Royal Marsden NHS Foundation Trust, London, United Kingdom; 2 CR UK Cancer Imaging Centre, The Institute of Cancer Research and The Royal Marsden NHS Foundation Trust, London, United Kingdom; 3 Department of Radiotherapy, The Royal Marsden NHS Foundation Trust, London, United Kingdom; 4 Medical Physics in Radiology, The German Cancer Research Center (DKFZ), Heidelberg, Germany; 5 Author to whom any correspondence should be addressed.; Martin.Leach@icr.ac.uk

**Keywords:** 4D MRI, radiotherapy treatment planning, MR-guided radiotherapy, pseudo CT, synthetic CT

## Abstract

MR-guided radiotherapy treatment planning utilises the high soft-tissue contrast of MRI to reduce uncertainty in delineation of the target and organs at risk. Replacing 4D-CT with MRI-derived synthetic 4D-CT would support treatment plan adaptation on hybrid MR-guided radiotherapy systems for inter- and intrafractional differences in anatomy and respiration, whilst mitigating the risk of CT to MRI registration errors.

Three methods were devised to calculate synthetic 4D and midposition (time-weighted mean position of the respiratory cycle) CT from 4D-T1w and Dixon MRI. The first approach employed intensity-based segmentation of Dixon MRI for bulk-density assignment (sCT_D_). The second step added spine density information using an atlas of CT and Dixon MRI (sCT_DS_). The third iteration used a polynomial function relating Hounsfield units and normalised T1w image intensity to account for variable lung density (sCT_DSL_). Motion information in 4D-T1w MRI was applied to generate synthetic CT in midposition and in twenty respiratory phases. For six lung cancer patients, synthetic 4D-CT was validated against 4D-CT in midposition by comparison of Hounsfield units and dose-volume metrics. Dosimetric differences found by comparing sCT_D,DS,DSL_ and CT were evaluated using a Wilcoxon signed-rank test (*p*   =  0.05).

Compared to sCT_D_ and sCT_DS_, planning on sCT_DSL_ significantly reduced absolute dosimetric differences in the planning target volume metrics to less than 98 cGy (1.7% of the prescribed dose) on average. When comparing sCT_DSL_ and CT, average radiodensity differences were within 97 Hounsfield units and dosimetric differences were significant only for the planning target volume D99% metric. All methods produced clinically acceptable results for the organs at risk in accordance with the UK SABR consensus guidelines and the LungTech EORTC phase II trial. The overall good agreement between sCT_DSL_ and CT demonstrates the feasibility of employing synthetic 4D-CT for plan adaptation on hybrid MR-guided radiotherapy systems.

## Introduction

Magnetic resonance guided radiotherapy (MRgRT) exploits the high soft-tissue contrast of magnetic resonance imaging (MRI) to improve treatment delivery in radiotherapy (Lagendijk *et al* 2014, Schmidt and Payne 2015). Computed tomography (CT) images can be registered to magnetic resonance (MR) images to optimise radiotherapy treatment planning (RTP); electron density information from the CT images are employed for dose calculations and MR images are used to facilitate target and organ at risk (OAR) delineation (Owrangi *et al* 2018). Registration can result in systematic errors that propagate through the workflow and have been reported to be 2–5 mm for the brain and prostate (Edmund and Nyholm 2017). Registration errors, between CT and MRI, can be eliminated by deriving synthetic CT (sCT) from MRI (Johnstone *et al* 2018).

In conventional radiotherapy workflows, treatment delivery is based on the same pre-treatment CT image for all fractions, which is problematic as the patient anatomy might change during the course of treatment, for instance because of tumour shrinkage. Dosimetric errors related to inter- and intrafractional differences in anatomy can be reduced when delivering adaptive MRgRT (Raaymakers *et al* 2009, Fallone 2014, Lagendijk *et al* 2014, Mutic and Dempsey 2014, Thwaites *et al* 2014, Kontaxis *et al* 2017). In this scenario, treatment plans can be updated with the online patient anatomy and position obtained using sCT acquired during a hybrid MRgRT treatment session. Calculation of sCT by registering the pre-treatment CT image to daily MRI acquired prior to each treatment fraction was demonstrated (Kraus *et al* 2017). However, generating sCT directly from MRI is more desirable because it would eliminate registration errors and simplify the radiotherapy workflow by reducing the total number of scans (Edmund and Nyholm 2017).

Alternatively, sCT has been calculated using bulk-density assignment, atlas-based, voxel-based (including machine learning) and hybrid methods, with the majority of approaches applied to relatively immobile sites, such as the brain or prostate (Edmund and Nyholm 2017, Johnstone *et al* 2018). In the abdominothoracic region, most current methods are based on tissue-segmentation and bulk-density assignment. Of the methods which do not include bone-density information, dosimetric differences of the D95% metric (dose delivered to at least 95% of the planning target volume (PTV)) between sCT and CT have been reported to be less than 1% using 3D conformal RTP (Jonsson *et al* 2010), and greater than 5% using volumetric modulated arc therapy (VMAT) RTP (Prior *et al* 2017). In methods which include bone-density information, for instance by using an anterior vertebral body model (VMAT RTP) (Bredfeldt *et al* 2017) or an atlas (intensity modulated RTP) (Wang *et al* 2017), mean dosimetric differences for all regions and metrics have been reported to be within 1%.

There is scope to improve abdominothoracic sCT. Prior *et al* (2017) demonstrated that incorrect bulk-density assignment in the lung leads to errors up to 19.6% in the PTV dose-volume metrics. Patient specific lung electron density values should therefore be implemented to account for underlying lung pathology (Rosenblum *et al* 1980, Soejima *et al* 2000, Durham and Adcock 2015). Furthermore, four-dimensional (4D) or midposition (MidP) (time-weighted mean position of the respiratory cycle) (Wolthaus *et al* 2008a) sCT might be employed to account for respiratory motion in dose reconstruction (Al-Ward *et al* 2018).

In this article, thoracic 4D/MidP-sCT was calculated using three different methods and validated dosimetrically against the corresponding MidP image of 4D-CT for plan adaptation on MRgRT systems. In the first method, 4D/MidP-sCT was obtained using tissue-segmentation and bulk-density assignment (sCT_D_), and was extended in the second method using an atlas to include bone-density information (sCT_DS_). The third method employed a fitting approach to account for variable lung density (sCT_DSL_).

## Materials and methods

### Data acquisition

Six patients with early stage non-small cell lung cancer (5 adenocarcinoma, 1 mixed adenosquamous carcinoma), all of whom were treated with stereotactic radiotherapy, were included in this study after giving written informed consent. A 4D-CT scan was obtained for all patients using a Brilliance Big Bore CT scanner at 120kV (Philips Medical Systems, Best, The Netherlands), with voxel-size (0.98  ×  0.98–1.17  ×  1.17)  ×  2 mm^3^ and ten respiratory phases. Within a median of 2 (range: 0–14) d, MRI was acquired at 1.5 Tesla (MAGNETOM Aera; Siemens Healthcare, Erlangen, Germany) using a golden-angle radial T1-weighted stack-of-stars spoiled gradient echo sequence (Block *et al* 2014) in free breathing and a Cartesian 2-point Dixon gradient echo sequence in exhalation. Patients unable to breath-hold were scanned in free-breathing with four averages, resulting in an image close to exhalation. Patients were scanned in the same treatment position in both MRI and CT acquisition, which was enabled using an MR compatible immobilisation board (Extended Wing Board; Civco Radiotherapy, Coralville, IA, USA). During MR acquisition, an in-house built body coil holder was used to prevent compression of the body contour by the 18-channel receive array. Detailed MRI acquisition parameters can be found in table 1.

**Table 1.** Detailed acquisition parameters. No, number; Acq, acquisition; NA, not applicable.

Parameter	Radial T1w stack-of-stars	Dixon (exhalation)	Dixon (free-breathing)
Orientation	Axial	Axial	Axial
No. slices	80–88	64–88	72–88
No. spokes	1005	NA	NA
Acq. time (min)	05:19–05:45	00:20–00:21	02:02–02:49
Field of view (mm^2^)	320 × 320–336 × 336	322 × 430–360 × 480	312 × 400–368 × 469
Voxel-size (mm^3^)	(1.25 × 1.25–1.31 × 1.31) × 3.5	(1.68 × 1.68–1.88 × 1.88) × 4.2	(1.25 × 1.25–1.34 × 1.34) × 3.5
Echo time (ms)	1.55	2.39, 4.77	2.39, 4.77
Repetition time (ms)	3.18	7.6	7.6
Flip angle (°)	8	8	8
Pixel bandwidth (Hz)	630	400	400
No. signal averages	1	1	4
Fat suppression	YES	NO	NO

### Reconstruction of T1w data

T1w MRI was reconstructed using the 4D joint motion-compensated high-dimensional total variation (4D joint MoCo-HDTV) algorithm (Rank *et al* 2017). Prior to reconstruction, the raw data were corrected with an adaptive gradient-delay compensation (Block and Uecker 2011) and sorted into 20 overlapping respiratory phases using a self-gating signal based on the *k*-space centre (Paul *et al* 2015). After reconstruction, a 3D geometrical distortion correction was applied to each respiratory phase to account for gradient non-linearity, using the spherical harmonic coefficients provided by the vendor (Doran *et al* 2005). More details regarding the reconstruction workflow can be found in Freedman *et al* (2017).

### Motion-modelling

**Figure 1.**
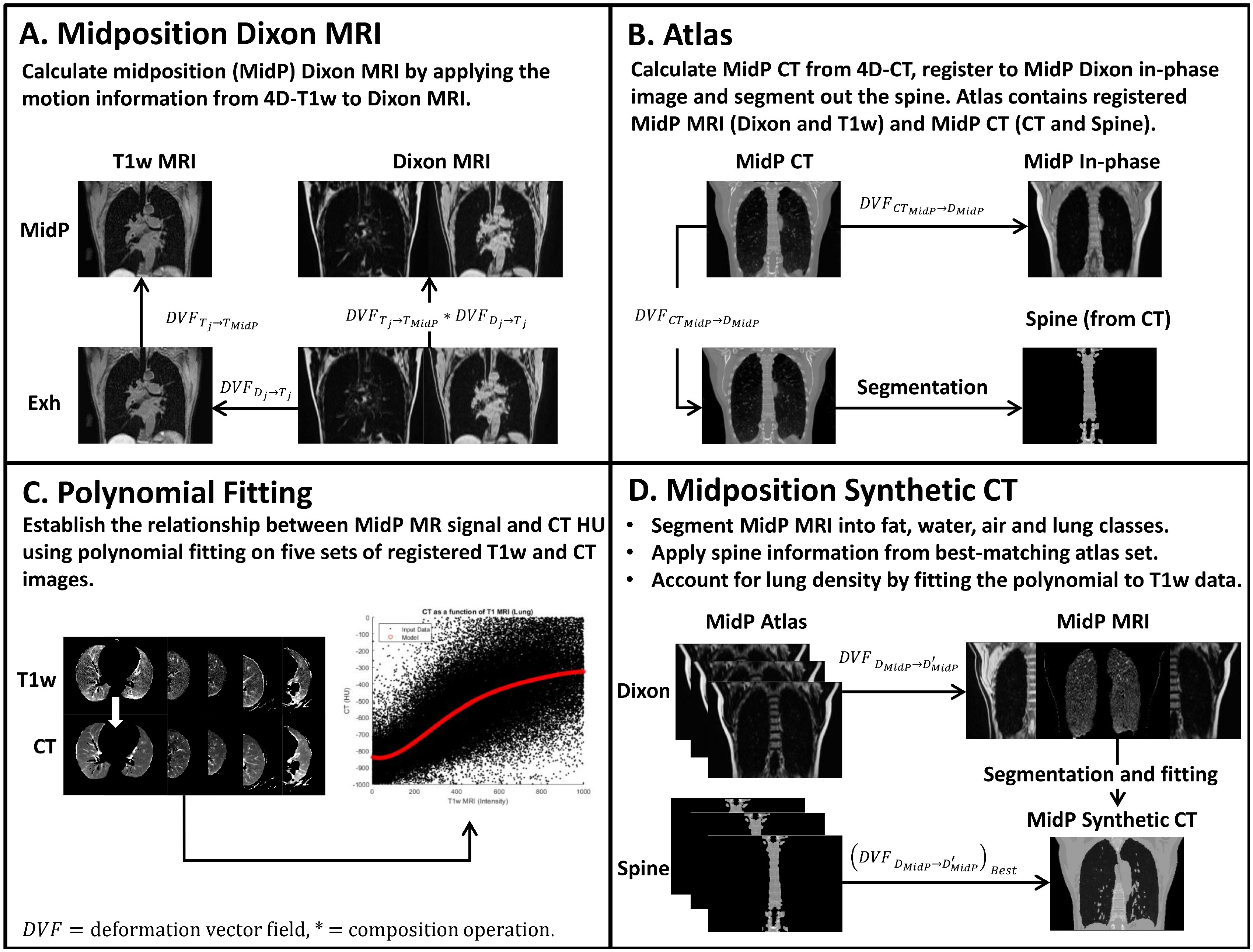
(A) The transformation from the *j* th respiratory phase to midposition (MidP) of 4D-T1w MRI is calculated (${\rm DV}{{{\rm F}}_{{{T}_{j}}\to {{T}_{MidP}}}}$). MidP-Dixon MRI is obtained by composing the DVF found by registering Dixon MRI to the *j* th respiratory phase of 4D-T1w MRI (${\rm DV}{{{\rm F}}_{{{D}_{j}}\to {{T}_{j}}}}$) with ${\rm DV}{{{\rm F}}_{{{T}_{j}}\to {{T}_{MidP}}}}$. (B) Describes the key steps involved in generating the atlas. (C) Describes polynomial fitting. The data used to ascertain the polynomial coefficients (5 patients) were obtained from the atlas. (D) Synthetic MidP-CT is calculated with a tissue-segmentation and bulk-density assignment method (sCT_D_), updated with non-rigidly registered spine-density information from the best-matching atlas image set (sCT_DS_), and variable lung density information using the fitting method (sCT_DSL_). ${\rm DV}{{{\rm F}}_{{{D}_{MidP}}\to D_{MidP}^{\prime }}}$ describes the transformation of each atlas MidP-Dixon (fat) MRI to the incoming MidP-Dixon (fat) MRI; ${{\left( {\rm DV}{{{\rm F}}_{{{D}_{MidP}}\to D_{MidP}^{\prime }}} \right)}_{Best}}$ describes transformation of the best-matching MidP-spine image to incoming MidP MRI.

All of the following calculations were carried out in MATLAB (version 2017a; The Mathworks, Natik, MA) on an Intel Xeon E5-1660 processor with 8 cores at 3 GHz and 64 GB of memory. Key components of the following motion-modelling method are provided in figure 1(A).

Based on volumetric normalised mutual information (NMI) (Pluim *et al* 2003), the closest matching respiratory phase of 4D-T1w MRI ($T{{1}_{j}}$) was chosen with respect to the in-phase Dixon image (${{D}_{j}}$). Deformation vector fields (DVFs) were calculated by non-rigidly registering $T{{1}_{j}}$ to all $n$ remaining respiratory phases $\left( {\rm DV}{{{\rm F}}_{{{T}_{j}}\to {{T}_{n}}}} \right)$. All b-spline GPU accelerated non-rigid registrations were carried out using NiftyReg (Modat *et al* 2010). A chain method concatenating DVFs was employed to reduce errors resulting from large deformations (Freedman *et al* 2017). The transformation ${\rm DV}{{{\rm F}}_{{{T}_{j}}\to {{T}_{MidP}}}}$ from the closest matching phase to MidP was determined from the ${\rm DV}{{{\rm F}}_{{{T}_{j}}\to {{T}_{n}}}}$ set (Wolthaus *et al* 2008a). MidP-T1w MRI was obtained by applying ${\rm DV}{{{\rm F}}_{{{T}_{j}}\to {{T}_{MidP}}}}$ to $T{{1}_{j}}$. ${\rm DV}{{{\rm F}}_{{{D}_{j}}\to {{T}_{j}}}}$ was generated by non-rigidly registering ${{D}_{j}}$ to $T{{1}_{j}}$. Water, fat and in-phase Dixon images were warped to MidP using the composition: ${\rm DV}{{{\rm F}}_{{{T}_{j}}\to {{T}_{MidP}}}}*{\rm DV}{{{\rm F}}_{{{D}_{j}}\to {{T}_{j}}}}$.

MidP-CT was independently calculated from 4D-CT in the same way that MidP-T1w MRI was obtained from 4D-T1w MRI.

### Atlas

The atlas contained MidP-T1w, MidP-Dixon (fat, water and in-phase), MidP-CT and MidP-spine (segmented from MidP-CT) images. The MidP-CT atlas images were obtained by registering the pre-calculated MidP-CT images to the MidP in-phase images. The MidP-spine atlas images were extracted from the MidP-CT atlas images using a thresholding and region of interest (ROI) method (figure 1(B)):

Bone was segmented from MidP-CT by thresholding (125 to 1500 Hounsfield units (HUs)) and connected component analysis; the first connected component corresponded to the rib cage, which included the thoracic vertebrae. Stray pixels were removed by morphologically dilating and closing the rib cage image using an ellipsoid structuring element (1,1,2 pixels). A rectangular ROI was manually placed around the spine on the central axial slice of the processed rib cage image. Pixels outside the ROI were set to zero on all slices. Holes were filled using a morphological flood-fill operation (Soille 1999).

A leave-one-out cross-validation was enabled by truncating the atlas to include all acquired data except the patient for which MidP-sCT was being generated (referred to as the incoming patient or image).

### Dixon synthetic CT (sCT_D_)

MidP-sCT (sCT_D_) was generated from the MidP-Dixon images using intensity-based segmentation and assignment of HUs for fat (−110), soft-tissue (70), air (−1000) and lung (−767). HU values were chosen from Wang *et al* (2017). Segmentation was carried out using binary masks:

A binary mask $M$ (background and lungs  =  1, remaining  =  0) was calculated by thresholding the summed MidP-Fat and MidP-Water images. Thresholds were set as the mean summed image intensity. $M$ was zero padded and subjected to connected component analysis; the largest component was the background mask $B$. The lung mask $L$ was calculated as $M-B$.

Fat and water masks were initialised by applying the $\left| 1-M \right|$ mask to the MidP-Fat and MidP-Water images. The initialised fat mask was thresholded using the mean non-zero intensity of the MidP-Fat image and then post-processed, to reduce stray pixels and holes, by keeping only the largest connected component. The post-processed fat mask was applied to remove fat components from the initialised water mask.

### Dixon-spine synthetic CT (sCT_DS_)

sCT_D_ were updated to include spine density information (sCT_DS_) from the best-matching MidP-spine atlas image. All MidP-Fat atlas images were registered to the incoming MidP-Fat image. The corresponding MidP-spine atlas images were warped with the resulting transformations and applied to segment the spine of registered MidP-Fat images. NMI was calculated between the incoming and registered segmented MidP-Fat images. The MidP-spine atlas image corresponding to the best-matching registered segmented MidP-Fat image (highest NMI) was fused with sCT_D_ by intensity override.

### Dixon-spine-lung synthetic CT (sCT_DSL_)

sCT_DS_ were modified to include variable lung density information (sCT_DSL_). The relationship between signal intensity in MidP-T1w images and HUs of co-registered MidP-CT images was modelled in the lung with a fifth order polynomial (figure 1(C)). MidP-T1w images were corrected for intensity inhomogeneity (Hofmann *et al* 2011): first the scanner-reconstructed T1w images were passed through 3D median and image guided filters (He *et al* 2013). MidP-T1w images were then divided by the filtered scanner-reconstructed images. Afterwards, inhomogeneity corrected MidP-T1w images were normalised such that the mean intensity values of the water components in each image were equal. Ten million non-zero points were randomly selected in the lungs of MidP-T1w MRI and MidP-CT. Data support over the entire intensity range was assured by further sorting the data into 20 equal sized bins, and then extracting 5000 random data points from each bin. The polynomial weights were then obtained by linear least squares fitting to the selected data.

In order to enable a leave-one-out cross-validation, the truncated MidP atlas (see Materials and methods: Atlas) data were used to calculate separate polynomial weights for each incoming patient. Variable lung density information was included by applying the fitted polynomial to incoming MidP-T1w intensity values. A summary of sCT_DSL_ generation can be found in figure 1(D).

For incoming patient 2 it was necessary to generate additional sCT images by linearly scaling the lung HU values of sCT_D,DS,DSL_ to match the median lung CT HU value (−865 HU). Scaling was required because patient 2 exhibited co-existent lung disease (severe emphysema) and did not fit into the group of other patients, which exhibited a mean (over patients 1 and 3–6) median HU value of  −808.

Four-dimensional sCT was calculated by warping MidP-sCT to all other respiratory phases using the composition of the ${\rm DV}{{{\rm F}}_{{{T}_{j}}\to {{T}_{n}}}}~$ and inverse ${\rm DV}{{{\rm F}}_{{{T}_{j}}\to {{T}_{MidP}}}}~$ transformations (for DVF calculations, see Materials and methods: motion-modelling).

### Synthetic CT: validation

MidP-sCT images were validated by comparison to MidP-CT, both dosimetrically and in terms of HUs. Radiation oncologists performed delineation and treatment planning. Using the information from non-rigidly registered MidP-T1w and MidP-Dixon images, one set of contours was generated for the primary tumour and OARs for each patient. The contours represented the ‘best fit’ between CT and MR images. Single full arc coplanar stereotactic VMAT plans at 6 MV were designed for all patients using a collapsed cone algorithm and an Agility multileaf collimator on RayStation (v5.99, RaySearch Laboratories, Sweden) with a dose grid voxel-size of 2.5 mm and collimator angle of 2°. Five patients had peripheral lesions with a PTV close to the chest wall, and were planned according to the UK SABR consortium guidelines with a five fraction regimen (55 Gy in 5 fractions) using the constraints stipulated in a recent UK SABR consensus publication (Hanna *et al* 2018). One patient had a central lesion and was planned using an eight fraction regimen (60 Gy in 8 fractions) as per the LungTech EORTC phase II trial protocol (Adebahr *et al* 2015, Lambrecht *et al* 2016). As MidP images were used, PTV margins were personalised for each patient, and this was dependent on tumour motion. PTV margins were calculated using motion information from the 4D-T1w images, but applying the same principles as reported elsewhere using 4D-CT planning (Wolthaus *et al* 2008b). Once contours had been finalised, they were copied onto the fused sCT images. Two planning techniques were used: initial planning on MidP-CT and independent re-calculation on sCT_D,DS,DSL_ (Plan 1); and initial planning on sCT_D,DS,DSL_ (using all sCT methods independently) and re-calculation on MidP-CT (Plan 2). Differences in the following dose-volume metrics were compared: Dose delivered to at least 95 or 99% of the PTV (PTV D95 or 99%, respectively), total PTV volume divided by the total isodose volume of interest (Conformity Index at Isodose), volume of lung receiving  ⩾20 Gy (v20 Gy), Proximal Airways near-point maximum dose (Dmax), which was defined as minimum dose to the 0.5 cm^3^ volume of the organ receiving the highest dose (Hanna *et al* 2018), Oesophagus Dmax 0.5 cm^3^, Brachial Plexus Dmax 0.5 cm^3^, Heart Dmax 0.5 cm^3^, Spinal Canal Dmax 0.1 cm^3^, and Chest Wall D 30 cm^3^, which is defined as the minimum dose to 30 cm^3^ of the organ that receives the highest dose. In an exploratory analysis, a Wilcoxon signed-rank test with a significance level of *p*   =  0.05 was applied to evaluate the dosimetric differences found by comparing sCT_D,DS,DSL_ and CT. Using the same analysis, the absolute values of the above-mentioned dosimetric differences were compared for significance between methods (sCT_D→DS,_ sCT_DS→DSL,_ sCT_D→DSL_).

## Results

**Figure 2.**
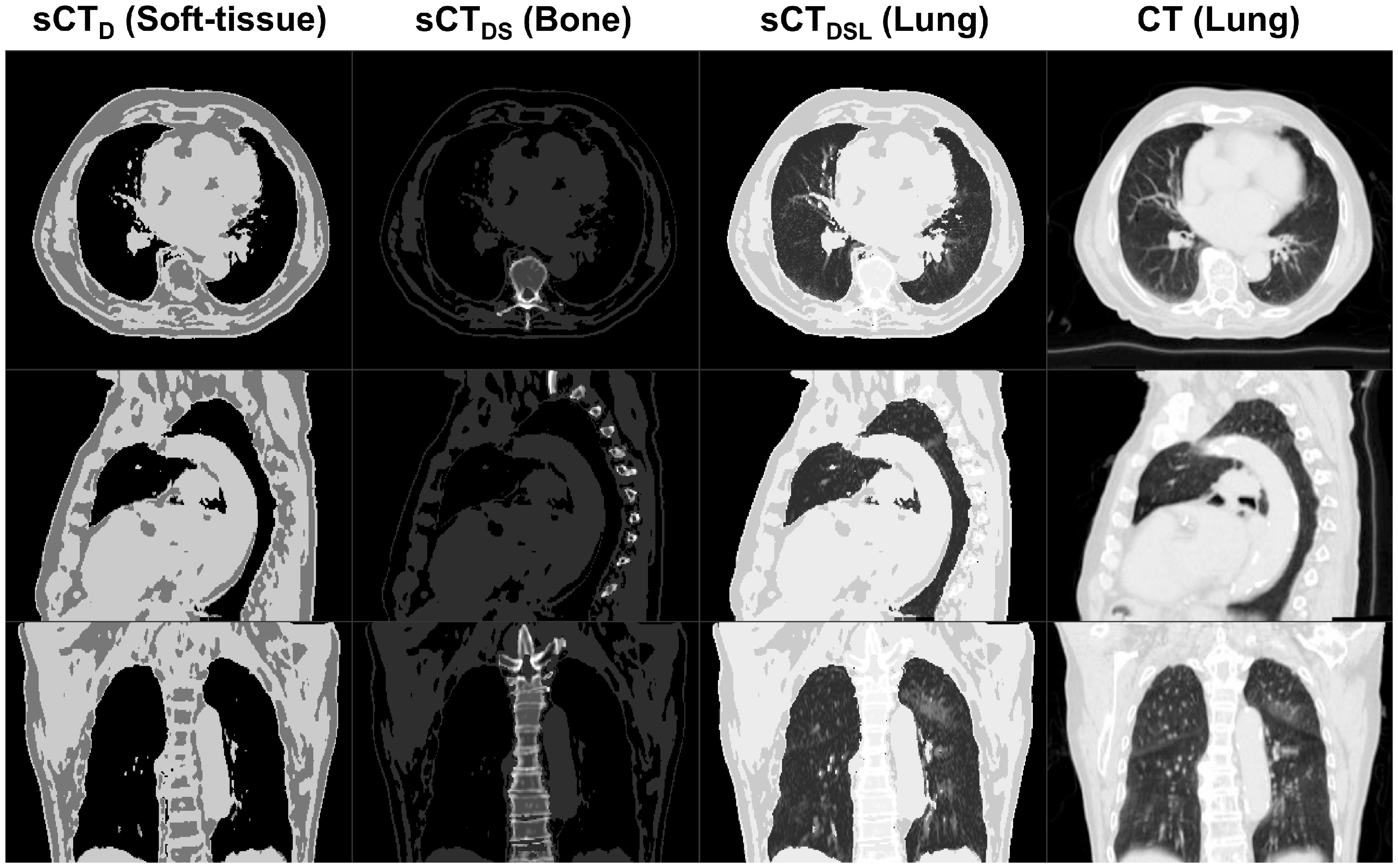
A comparison of Dixon (sCT_D_), Dixon-spine (sCT_DS_) and Dixon-spine-lung (sCT_DSL_) synthetic CT to midposition CT for patient 4. sCT is windowed (soft-tissue/bone/lung) to optimally display the new information that was introduced in each consecutive method.

For six patients with early stage node negative primary lung malignancies, three variations of MidP-sCT (sCT_D,DS,DSL_) were calculated. For these patients, mean PTV volume was 34 (range: 22–40) cm^3^. Four-dimensional T1w MRI reconstruction took between 9 and 12 h, and calculation of sCT_D_, sCT_DS_ and sCT_DSL_ was finished in 30, 51 and 54 min, respectively. Figure 2 shows an example reconstructed MidP-CT compared to sCT_D,DS,DSL_. sCT_DSL_ provided good visual agreement with MidP-CT, due to not only comparable spine and variable lung density, but also matching respiratory phase. Two example movies of 4D-sCT (sCT_DSL_, 20 respiratory phases) for patients 1 and 4 are provided as supplemental material (stacks.iop.org/PMB/64/115005/mmedia).

### Validation: Hounsfield units

**Figure 3.**
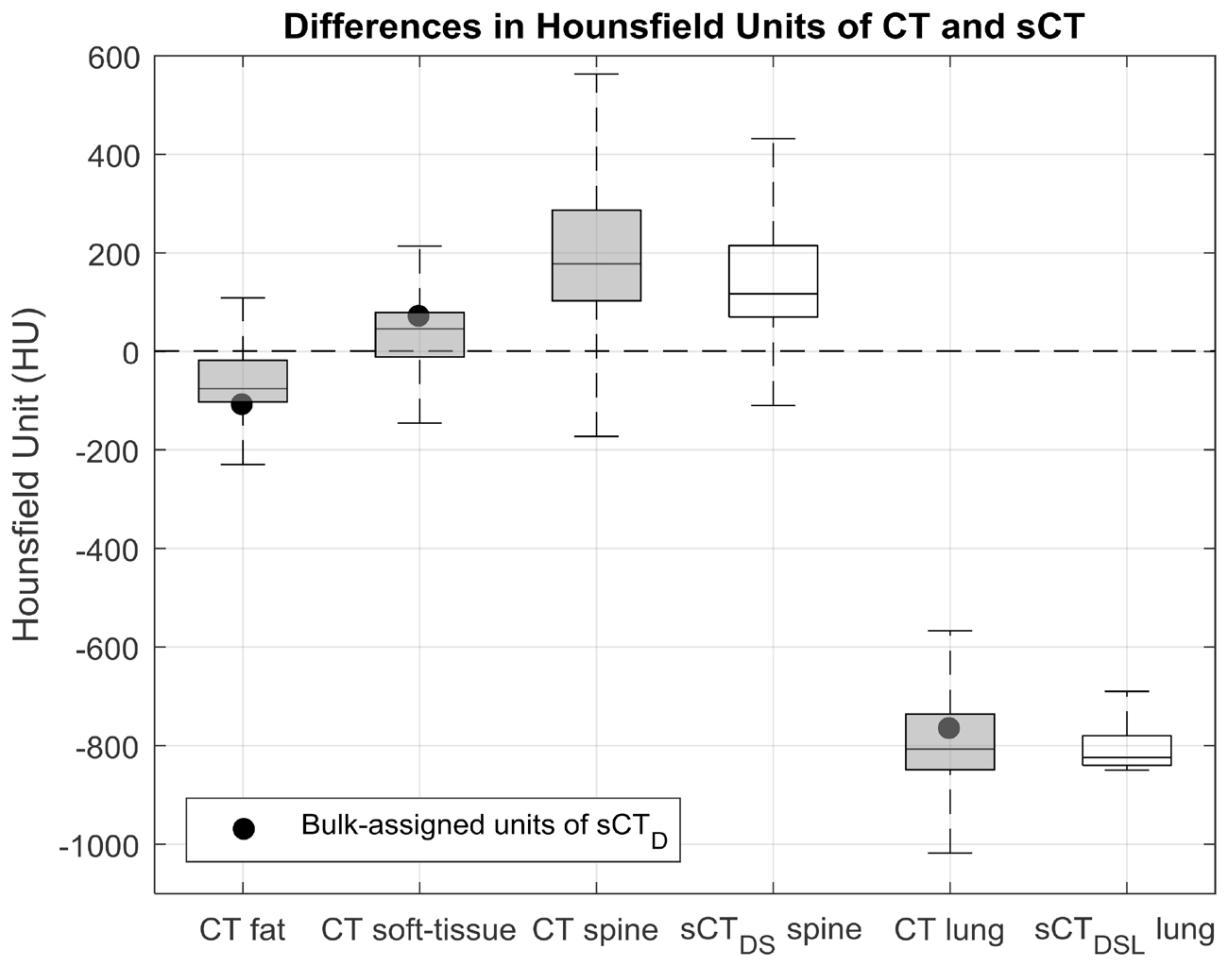
Regional comparison of Hounsfield units (HUs) in CT and synthetic CT (sCT) images. Boxplots summarise the HU distributions of each segmented region (fat, soft-tissue, spine and lung) for all patients; shading distinguishes between data obtained from CT (shaded) and sCT (unshaded) images. Filled black circles depict the bulk-assigned HU values of sCT_D_. Outliers in the data, determined as 1.5 times the interquartile range above the upper quartile or below the lower quartile, were removed for display. Due to the variability in both the spine and lung densities, HU values of CT best matched sCT_DSL_.

The median and standard deviation of the absolute differences in HUs, over all patients, between CT and sCT of the Dixon and Dixon-spine-lung methods were: 43  ±  187 and 42  ±  188 for the soft-tissue region, 43  ±  146 and 40  ±  144 for the fat region, 71  ±  114 and 43  ±  106 for the lung region, and 174  ±  186 and 96  ±  161 for the spine region; demonstrating that overall sCT_DSL_ exhibited the highest similarity with the ground-truth CT images (figure 3).

### Validation: dose-volume metrics

**Figure 4.**
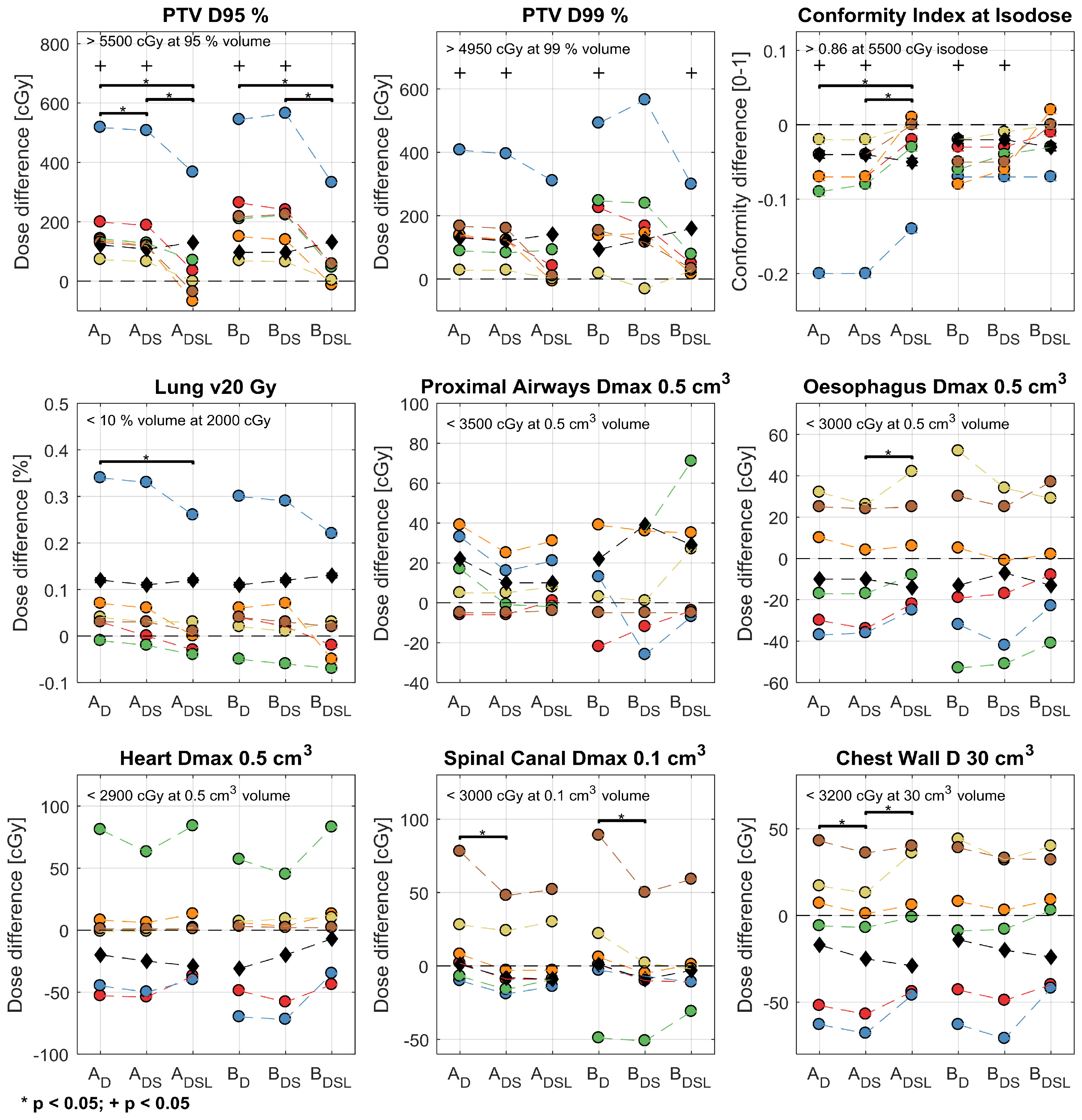
Differences in selected dose-volume metrics between the three methods of synthetic CT (sCT) and CT. *A*_D,DS,DSL_ denotes planning on CT and re-calculating on the sCT_D,DS,DSL_ images (Plan 1). *B*_D,DS,DSL_ codifies planning on the sCT_D,DS,DSL_ images and re-calculating on CT (Plan 2). Coloured circles encode patient number (red, blue, green, orange, yellow and brown codify patients 1 to 6, respectively). Black diamonds show the results of re-planning patient 2 (blue) with rescaled lung HUs. Black crosses show significant differences between sCT and CT; brackets and stars label significant absolute differences between sCT methods. Mandatory clinical goals for the five patients planned with 55 Gy in 5 fractions are listed on the top left-hand side of each subplot.

As presented in figure 4, differences in selected dose-volume metrics between sCT_D,DS,DSL_ and CT were significant for PTV metrics, but not for OAR metrics. Unlike sCT_D_ and sCT_DS_, sCT_DSL_ only exhibited a significant difference for the PTV D99% metric (*p*   =  0.03) of Plan 2 (planned on sCT and re-calculated on CT). For the PTV D95% and D99% metrics, sCT_DSL_ exhibited the lowest absolute difference with CT, which was (mean  ±  standard deviation in %) 1.7  ±  2.5 and 1.4  ±  2.3 for Plan 1 (initially planned on CT and re-calculated on sCT), and 1.6  ±  2.4 and 1.7  ±  2.2 for Plan 2. The mean and standard deviations were skewed by the results of patient 2, which exhibited absolute differences up to 6.7%. If the lung HU values of patient 2 were linearly scaled to match the median lung HU value of CT (figure 4; black diamonds), the average absolute differences of the PTV D95% and D99% metrics were reduced to: 0.91  ±  0.89 and 0.77  ±  1.1 for Plan 1, and 0.77  ±  0.93 and 1.0  ±  1.1 for Plan 2. The average absolute differences (over all patients) of the investigated dose-volume metrics between sCT_DSL_ and CT are summarised in table 2. For the OAR dose-volume metrics, minor differences were observed, but did not violate pre-defined clinical goals.

**Table 2.** The mean and standard deviation absolute differences (abs diff) between the Dixon-spine-lung sCT method (sCT_DSL_) and CT for the: Spinal Canal Dmax 0.1 cm^3^, Lung v20 Gy, Proximal Airways Dmax 0.5 cm^3^, Oesophagus Dmax 0.5 cm^3^, Brachial Plexus Dmax 0.5 cm^3^, Heart Dmax 0.5 cm^3^, Chest Wall D 30 cm^3^, Conformity Index at Isodose, PTV D95% and PTV D99% dose-volume metrics. Differences were taken with respect to CT for both Plan 1 (planned on CT and re-calculated on sCT_DSL_) and Plan 2 (planned on sCT_DSL_ and re-calculated on CT), and did not include the rescaled sCT of patient 2.

Metric	Plan 1 (abs diff)	Plan 2 (abs diff)	Plan 1 (% diff)	Plan 2 (% diff)
Spinal Canal	20 ± 18 (cGy)	19 ± 22 (cGy)	1.2 ± 0.8	1.2 ± 1.1
Lung v20 Gy	0.06 ± 0.10 (%)	0.07 ± 0.08 (%)	1.1 ± 1.7	1.3 ± 16
Proximal Airways	11 ± 12 (cGy)	25 ± 26 (cGy)	2.2 ± 3.3	3.1 ± 3.9
Oesophagus	21 ± 13 (cGy)	23 ± 16 (cGy)	1.3 ± 0.6	1.3 ± 0.8
Brachial Plexus	10 ± 17 (cGy)	8 ± 16 (cGy)	1.9 ± 2.9	1.4 ± 3.4
Heart	30 ± 32 (cGy)	31 ± 30 (cGy)	1.7 ± 1.0	3.0 ± 2.7
Chest Wall	29 ± 20 (cGy)	28 ± 17 (cGy)	1.0 ± 0.7	1.0 ± 0.6
Conformity Index	0.03 ± 0.05 (0–1)	0.02 ± 0.03 (0–1)	3.9 ± 6.3	2.5 ± 2.8
PTV D95%	96 ± 135 (cGy)	85 ± 123 (cGy)	1.7 ± 2.5	1.6 ± 2.4
PTV D99%	77 ± 119 (cGy)	84 ± 108 (cGy)	1.4 ± 2.3	1.7 ± 2.2

### Comparison of synthetic CT methods

Significant reductions in absolute differences were found for the PTV D95% (sCT_D→DS_, sCT_DS→DSL_ and sCT_D→DSL_) and Conformity Index at Isodose metrics (sCT_DS→DSL_ and sCT_D→DSL_) of Plan 1, and for the PTV D95% (sCT_D→DSL_ and sCT_DS→DSL_) and D99% metrics (sCT_DS→DSL_) of Plan 2. For all OAR metrics, except the Proximal Airways Dmax 0.5 cm^3^ metric, the mean absolute dosimetric difference over all patients decreased between sCT_D_ and sCT_DSL_. No significant absolute differences were found for the OAR metrics.

**Figure 5.**
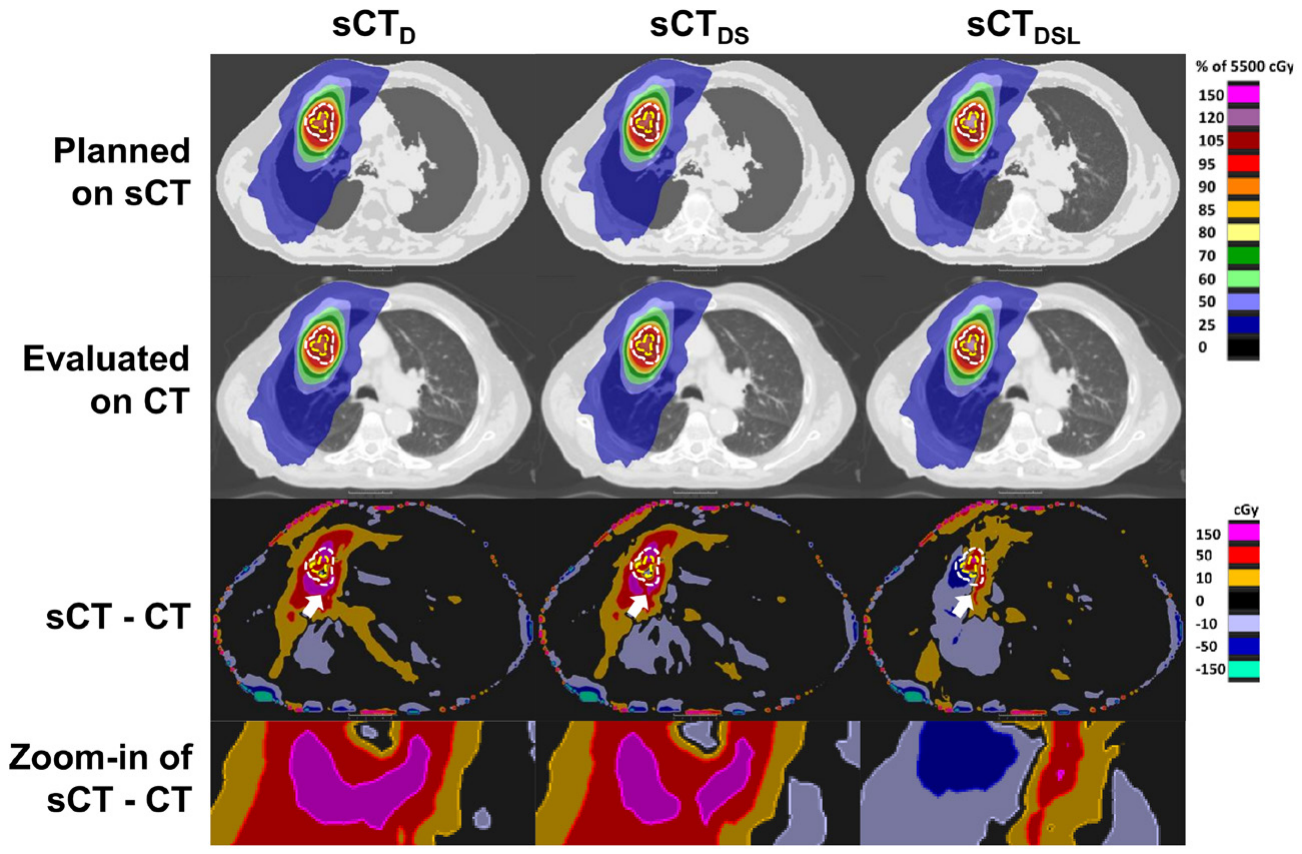
An example dosimetric comparison as planned on each of the three midposition synthetic CT images (sCT_D,DS,DSL_) and evaluated on the midposition CT (patient 4). First and second rows: simulated dose planned on sCT and re-evaluated on CT. Third row: differences between the first and second rows. The planning target and gross tumour volumes are contoured in dashed white and yellow lines. White arrows assist viewing of an example hot spot reduction when comparing successive sCT methods. Fourth row: zoom-in of the region indicated by the white arrows. Overall, differences in the simulated dose-distributions are reduced with consecutive sCT method.

Figure 5 displays an example illustrating the differences between the three sCT methods. Compared to sCT_D_, inclusion of spine density information in sCT_DS_ resulted in a reduction of local hot spots in the differences between the simulated dose distributions of sCT and CT. Inclusion of variable lung density in sCT_DSL_ led to a further reduction in dose differences. In particular, the appearance of hot spots around the PTV decreased.

## Discussion

In this article, three methods to calculate 4D-sCT were introduced and validated in MidP against 4D-CT. Employing 4D/MidP-sCT on hybrid MRgRT systems would enable plans to be adapted for anatomical differences and changes in respiratory pattern throughout the course of radiotherapy treatment, which might permit target dose boosting and sparing OARs (Al-Ward *et al* 2018), whilst mitigating the risk of registration errors between CT and MRI. Moreover, 4D-sCT could be combined with motion information from fast 2D cine MRI to obtain a patient-specific motion-model (Stemkens *et al* 2016), which might be applied to generate low-latency volumetric sCT. In the comparison of HUs and dose-volume metrics sCT_DSL_ exhibited the greatest agreement with MidP-CT, which was due to inclusion of variable spine and lung density. For Plan 2, the most clinically relevant scenario (planned on sCT and evaluated on CT), no significant differences were found between dose-volume metrics of sCT_DSL_ and CT, except for the PTV D99% metric. Furthermore, absolute differences between sCT_DSL_ and CT for the PTV D95% and D99% metrics were on average less than 1.7%. For sCT_DSL_, dose to OAR metrics varied over patients, but did not violate pre-defined clinical goals. The overall agreement suggests that sCT_DSL_ would enable plan adaptation on hybrid MRgRT systems for lung cancer patients. Further work is required to improve the methodology for patients with underlying pathology causing high variability in lung density.

Compared to sCT_D_, employing sCT_DS_ resulted in a reduction of the median and standard deviation HU errors in the spine by 78 and 25 HUs. Furthermore a significant dosimetric error reduction in the PTV D95% metric was obtained between sCT_D_ and sCT_DS_ for Plan 1. Inclusion of variable spine density was shown to decrease local hot spots in the differences between the simulated dose distributions of CT and sCT (figure 5), which might explain the reductions in HUs and dose-volume metric values. A reduction in the median and standard deviation lung HU error of 28 and 8 HUs was calculated between sCT_D_ and sCT_DSL_, which was complemented by a significant reduction of absolute dose differences in the PTV metrics for Plans 1 and 2. The reported sensitivity of the PTV metrics to the assigned lung HUs was corroborated by Prior *et al* (2017), who showed that the difference in the PTV D95% metric can vary up to 9.06% (target population average) when assigning bulk lung electron densities between 0.1–0.5 g cm^−3^.

For patient 2, lung HU values of sCT_D,DS,DSL_ were scaled to match the median lung HU value of CT. For the PTV D95% metric, scaled sCT_D_ and sCT_DSL_ displayed absolute differences of: 122 and 130 cGy for Plan 1; 97 and 132 cGy for Plan 2. These differences were lower than corresponding unscaled values for sCT_D_ and sCT_DSL,_ which were: 518 and 367 cGy for Plan 1; 544 and 332 cGy for Plan 2 (figure 4). On closer imaging review, patient 2 had severe emphysema—a disease which causes destruction of the alveolar septa, leaving enlarged air spaces and a loss of elastic recoil (Longmore *et al* 2014) (see supplemental material). Large differences in PTV dose-volume metrics might be explained by low signal-to-noise ratio in the lung, which might have led to incorrect assignment of enlarged air spaces as lung tissue. As demonstrated for patient 2, scaling of lung HU values to the average value on an available CT scan might be employed to correct for enlarged air spaces, without introducing registration errors. If no CT scan existed, as in a strict MR-only workflow, ultra-short echo time (Ohno *et al* 2016) or balanced steady-state free-precession sequences (Bauman *et al* 2009) could be employed to distinguish emphysema from healthy lung tissue.

Dosimetric accuracy of sCT might be affected by scanner and patient-dependent geometrical distortions. Patient-dependent distortions are caused by off-resonance due to magnetic susceptibility differences between tissues and chemical shift (Weygand *et al* 2016). Stanescu *et al* (2012) simulated the maximum susceptibility-induced field inhomogeneity in the thorax as 5.6 ppm, which corresponds for our acquisition to a maximum distortion of 0.90 pixel in the Dixon images (pixel bandwidth  =  400 Hz). In the T1w images, patient-dependent distortions manifest as blurring, due to the radial readout. The impact of minor patient-dependent distortions in the Dixon images was mitigated by non-rigidly registering to the radial T1w images. Alternatively, patient-dependent distortions could be corrected for using separately acquired B0 maps. We corrected for scanner-dependent gradient non-linearity induced distortions, but expect residual distortions, which increase in magnitude with distance from isocenter (Doran *et al* 2005). Huang *et al* (2016) reported average residual distortion errors within 1.5 mm over radial distances up to 200 mm from isocenter. Accuracy of sCT, in particular the body contour, is therefore subject to the specified tolerances by the vendor. We minimised the impact of residual geometrical distortions in our validation study by registering MRI to CT.

We have presented a precise methodology to generate 4D/MidP-sCT which is promising for PTV metrics and clinically acceptable for OAR metrics. However, our approach has several drawbacks. Due to the time factor involved in reconstruction of 4D-T1w MRI, it is not yet feasible for application within the same session as acquired; currently limiting its applicability for MRgRT systems. The estimation of DVFs based on the 4D-MRI makes our 4D-sCT method independent of the MR image reconstruction algorithm. For instance, reconstruction time of 4D-MRI might be reduced to a clinical timeframe of 5–10 min using a state-of-the-art server (Mickevicius and Paulson 2017). Because of its ease of implementation, a best-atlas method was employed for multi-atlas label-fusion when performing spine segmentation. Alternative label-fusion techniques, such as majority voting (Iglesias and Sabuncu 2015, Kieselmann *et al* 2018) or a two-step local weighting method (Arabi *et al* 2016), might provide dosimetric improvements related to spine density. One limitation of our study is that the static B0 magnetic field was not accounted for during treatment planning, which will result in dosimetric uncertainties associated with the electron return effect (Raaijmakers *et al* 2005). Menten *et al* (2016) compared conventional lung stereotactic treatment plans with and without the magnetic field and reported significant dosimetric differences only for the skin OAR, which would suggest that our results remain applicable to MRgRT.

To our knowledge this is the first time that 4D-MRI has been used to calculate and verify sCT in the midposition of the respiratory cycle. Employing the MidP image results in similar planning margins to idealised gated radiotherapy (Wolthaus *et al* 2008a). Our method also supports generation of 4D-sCT (supplemental material: Movies 1 and 2); enabling alternative planning images, such as the mid-ventilation image (closest respiratory phase to MidP), to be calculated (Wolthaus *et al* 2006, 2008b). In Prior *et al* (2017), an incorrect lung HU bulk-assignment was shown to cause errors larger than 5% in the PTV dose-volume metrics. To optimise lung HU assignment, we devised a polynomial fitting method to account for variable lung density, which significantly reduced errors in the PTV dose-volume metrics. The polynomial fitting method is sensitive to intensity inhomogeneity resulting from non-uniform receiver coil profiles. We addressed this problem by implementing an intensity correction based on the vendor-provided image normalisation. A fifth order polynomial was chosen because it well represented the function returned using Gaussian Process Regression (Freedman *et al* 2018), but was faster to train and apply to incoming data. In the presented work, the polynomial weights and spine density information were calculated from truncated atlas data, which enabled a leave-one-out cross-validation. In clinical practice, the same atlas would be employed for all incoming patients. In Wang *et al* (2017), absolute mean errors in the PTV dose-volume metrics were reported to be less than 1%. However in Wang *et al* (2017), lung cancer was simulated using homogeneous spherical lung lesions in mostly non lung cancer patients. Due to the strong relationship between lung cancer and underlying lung pathology (Durham and Adcock 2015), it is possible that the low errors reported in Wang *et al* (2017) are not fully representative of actual lung cancer patients, which were the target population in our study.

## Conclusion

Three methods to calculate 4D-sCT were developed and validated on six lung cancer patients by comparison with 4D-CT using HUs and dose-volume metrics in the midposition of the respiratory cycle. Compared to bulk-density assignment, inclusion of variable spine and lung density led to significantly reduced dosimetric differences in PTV metrics. For sCT generated using the Dixon-spine-lung method, dosimetric differences were clinically acceptable for OAR metrics, and they were on average  ⩽98 cGy (1.7%) for PTV metrics. We have demonstrated the feasibility of calculating thoracic 4D-sCT from 4D-T1w and Dixon MRI for treatment plan adaptation on hybrid MRgRT systems.
